# Letter to the editor regarding “Accelerated versus standard initiation of renal replacement therapy for critically ill patients with acute kidney injury: a systematic review and meta-analysis of RCT studies”

**DOI:** 10.1186/s13054-021-03528-2

**Published:** 2021-03-09

**Authors:** Kate Magner, Edward Clark, Swapnil Hiremath

**Affiliations:** 1 Faculty of Medicine, The University of Ottawa, 451 Smyth Road, Ottawa, ON K1H8M5 Canada; 2 The Ottawa Hospital, 501 Smyth Road, Ottawa, ON K1H8L6 Canada; 3 The Ottawa Hospital, Riverside Campus, 1967 Riverside Dr., Ottawa, ON K1H7W9 Canada

To the Editor

On the heels of the Standard versus Accelerated Initiation of Renal-Replacement Therapy in Acute Kidney Injury (STARRT-AKI) trial [1], Pan et al. [2] recently published a systematic review and meta-analysis of randomised controlled trials (RCTs) on the timing of renal replacement therapy for critically ill patients with acute kidney injury. They concluded that accelerated dialysis might benefit survival in a subgroup of surgical ICU patients, but not in a mixed ICU population. They also found that early initiation might have benefited patients who received CRRT, but not those who received other dialysis modalities. These results are based on subgroup analyses that included trials conducted solely in surgical ICU settings, or, in the second instance, where CRRT was the only type of dialysis used. The two trials conducted in surgical ICU settings, Sugahara et al. [3] and Zarbock et al. [4], included only 28 and 231 patients, respectively. In contrast, the STARRT-AKI trial, which was not factored into this analysis, included an overall population of 2927 patients of whom 965 were surgical patients. We determined that if these surgical patients had been included in the subgroup analysis, it would have resulted in a null effect for early initiation (OR 0.96, 95% CI 0.77, 1.21). Likewise, a subgroup analysis of trials involving only CRRT ignores that approximately 70% of the patients in the STARRT-AKI received CRRT as the initial RRT modality. Lastly, we note the omission of another potentially eligible trial, from Jamale et al. [5], which also reported no benefit with accelerated renal replacement therapy. On the whole, omitting the surgical or CRRT subgroups from the trials done in mixed ICU populations, particularly those from the STARRT-AKI trial, renders fallible the conclusion that accelerated renal replacement therapy initiation might reduce all-cause mortality in these settings. An individual patient-level meta-analysis might be more informative, but otherwise, the results of the largest trial, namely STARRT-AKI, are more robust than subgroup analyses that only considered patients from underpowered trials.

## Data Availability

Data sharing not applicable to this article as no datasets were generated or analysed during the current study.
